# *MAOA* Influences the Trajectory of Attentional Development

**DOI:** 10.3389/fnhum.2016.00424

**Published:** 2016-08-25

**Authors:** Rebecca A. Lundwall, Claudia G. Rasmussen

**Affiliations:** Development of Visual Cognition Laboratory, Department of Psychology, Brigham Young UniversityProvo, UT, USA

**Keywords:** longitudinal, reflexive attention, infants, children, early identification, intervention

## Abstract

Attention is vital to success in all aspects of life (Meck and Benson, 2002; Erickson et al., 2015), hence it is important to identify biomarkers of later attentional problems early enough to intervene. Our objective was to determine if any of 11 genes (*APOE, BDNF*, *HTR4, CHRNA4, COMT, DRD4, IGF2, MAOA*, *SLC5A7*, *SLC6A3*, and *SNAP25*) predicted the trajectory of attentional development within the same group of children between infancy and childhood. We recruited follow up participants from children who participated as infants in visual attention studies and used a similar task at both time points. Using multilevel modeling, we associated changes in the participant’s position in the distribution of scores in infancy to his/her position in childhood with genetic markers on each of 11 genes. While all 11 genes predicted reaction time (RT) residual scores, only Monoamine oxidase A (*MAOA*) had a significant interaction including time point. We conclude that the *MAOA* single nucleotide polymorphism (SNP) rs1137070 is useful in predicting which girls are likely to develop slower RTs on an attention task between infancy and childhood. This early identification is likely to be helpful in early intervention.

## Introduction

Because attention is vital to success in all aspects of life (Meck and Benson, 2002; Erickson et al., 2015), it is important to identify biomarkers of later problems with attention early enough to intervene. Doing so may alleviate the development of attentional problems in psychological disorders such as attention-deficit disorder (ADHD; Abed, 2014), anxiety (Bar-Haim et al., 2011), autism spectrum disorders (Camarata, 2014), and depression (Mueller et al., 2012). Longitudinal studies are especially important because they allow us to determine which early symptoms actually lead to later problems. Making an early identification and intervening are consistent pursuits among those researching developments (Moss et al., 1982; Webb and Jones, 2009; Camarata, 2014; DuPaul and Stoner, 2014). Early identification is possible with some biomarkers.

Genes are a logical choice for early biomarkers because they control the availability of neurotransmitters. The biological pathway from a particular gene to attentional behavior is often hypothesized to include interference with normal neural messaging across a synapse as neurotransmitter availability is affected. The varying availability of these neurotransmitters due to genetic differences could make behaviors like attending less efficient in some individuals, and this, in turn, could be captured by various measures of attention, including outcomes from computer tasks. Based on observing associations between specific genes and early visual attention, some researchers have called for studies on the developmental course of genotypic differences from infant attention (Holmboe et al., 2010).

Identifying genetic influences on the development of attentional deficits will allow interventions to be created. However, first we must identify early individual differences in behavior to which genetic differences can be associated. One aspect of attention that can be studied in infancy is reflexive attention. Reflexive attention is a component of overall attention that deals with the stimulus-driven information processing. It can be contrasted to sustained attention, the other component of attention. Some studies suggest that reflexive and sustained attention are independent components of overall attention (Barry et al., 2001; Berger et al., 2005; Andrade et al., 2009; Underbjerg et al., 2013; Dye and Hauser, 2014).

Studies using habituation and paired comparisons (Fantz, 1961; Fagan, 1970; Bornstein and Sigman, 1986) have found that infant measures of information processing can predict child information processing (Fagan and Singer, 1983; Rose and Wallace, 1985; Dougherty and Haith, 1997; Rose and Feldman, 1997; Rose et al., 2003; Kavsek, 2004). Note that information processing measures are often more related at the two time points than infant IQ (which relies on motor skills) and child IQ (which relies on cognitive skills; Colombo, 1993; McCall, 1994; Sigman et al., 1997; Tucker-Drob et al., 2013).

For our information processing task, we use a forced-choice preferential looking task with a left or right moving bar in a field of static bars (Dannemiller, 1998). When movement captures attention, there is “selection” of the moving bar from among the other static bars on the display and the infant looks at the moving bar. This task provides percent correct (PC) and latency (RT) for the adult observer to make left-right looking judgments that reflect the speed and clarity of signals the infant sends (Teller, 1997).

We could find no studies using infant forced-choice preferential looking to predict future cognitive outcomes. The task has, however, been used to classify toddlers with autism or developmental delays (Pierce et al., 2011). This suggests that preferential looking may be useful to predict risk for future attention related disorders. Our interest in reaction time (RT) trajectory is to determine at an early age, which children might develop problems with attention and need intervention. Such results would be the first to our knowledge to demonstrate genetic influence on the trajectory of the development of reflexive attention.

Several genes have been identified as associated with poor attention at one point or another during development (Frank et al., 2004; Brookes et al., 2006; Ribasés et al., 2008; English et al., 2009; Nobile et al., 2010; Bidwell et al., 2011; Nymberg et al., 2013; Gálvez et al., 2014). Dopamine genes are often associated with attentional deficits (Smith et al., 2003; Li et al., 2006; Genro et al., 2008; Holmboe et al., 2010).

In addition to dopaminergic genes, other genes are plausibly associated with attentional development. For example, *APOE*, *SLC5A7*, and *CHRNA4* are all associated with the neurotransmitter acetylcholine, which has been associated with cognitive development (McKinnon and Nathanson, 1995) and with visuospatial attention, ADHD, and distractibility (Manuck et al., 2000; Manor, 2002; Störmer et al., 2012; Markant et al., 2014). Likewise, *BDNF*, *HTR4*, and Monoamine oxidase A (*MAOA*) are associated with serotonin, although *MAOA* and *BDNF* also influence dopamine availability (Yu et al., 2005; Razgado-Hernandez et al., 2015; Voigt et al., 2015; Parikh et al., 2016). Serotonin has been associated with brain and attentional development (Binder and Scharfman, 2004; Shim et al., 2008; Faraone and Mick, 2010) and with ADHD (Poirier, 1996; Greenwood et al., 2000; Fisher et al., 2002; Parasuraman et al., 2002; Walitza et al., 2005; Winterer et al., 2007). Overall, the likelihood of genetic influence on the trajectory of attentional development is high because these genes influence such developmental processes as axon guidance, neuronal cell mobility, synaptic function, and chromosomal remodeling (Gilman et al., 2012; Douet et al., 2014). While the “snapshot” associates described above are useful, it would be even more useful to be able to predict the likelihood of declining attentional performance over development to determine who might most need intervention to prevent the development of more serious problems.

Trajectory research is essentially a longitudinal research with an emphasis on individual differences. Studying the trajectory of specific outcomes based on earlier risk factors has been especially important in health research (Henly et al., 2011). A recent study used the concept of trajectory of development and determined that children with a Williams Syndrome diagnosis are not simply delayed in development, but show a distinctly different path of development (Annaz et al., 2009). Other researchers have found trajectory research useful in studying genetic influences on cognitive development (Torgersen, 1981; Plomin et al., 1993; Sigman et al., 1997; Tucker-Drob et al., 2013). Because it tracks the same children at two or more time points (Farrington, 1991; Selig and Little, 2012; Grammer et al., 2013), trajectory research could indicate if genetic variation is associated with developmental change in attention. Such influences on development are plausible because genes influence neuronal and synaptic changes from infancy to childhood.

In particular, we are asking whether genes influence the trajectory of reflexive attention development. To answer this question, we recruited participants from a follow up sample of children who participated as infants in visual attention studies (Dannemiller, 2004). We use RT and PC to index performance on a reflexive attention task. Better reflexive attention orients more quickly and accurately to a moving bar.

## Materials and Methods

To determine if genetic variation is associated with developmental change in attention, we selected genetic markers based on their biological and functional effects. There are a very large number of genes related to brain development and the function of the majority of these genes is poorly understood (Dixon-Salazar and Gleeson, 2010). Exploring the influence of several candidate genes with a plausible biological influence on attention to determine if that influence alters the trajectory of development can be useful. We focused our selection on genes that had already been mentioned in the literature as related to some aspect of attention, cognition, growth, or brain development and that had a plausible biological explanation for their potential influence on reflexive attention. For example, some genes were selected for study because they are related to growth generally. Others were selected because they are related to brain growth more specifically. Insulin-like genes are one example of a growth-related gene and there has been some suggestion that these may also influence cognition (Borenstein et al., 2006). In addition, *APOE* is highly expressed during development and is associated with both epilepsy and schizophrenia (Ziats and Rennert, 2011). Another way to select genes related to brain development is by examining the stage at which the gene is likely to affect cortical development. For example, defects in genes involved in controlling neurogenesis are likely to cause more severe brain disorders (such as autosomal recessive primary microcephaly, which involves reduced neuronal numbers) than genes that control the developmentally later process of circuit formation (such as *CHRNA4* associated with epilepsy; Dixon-Salazar and Gleeson, 2010). *MAOA* is a gene on the X chromosome whose gene product is a mitochondrial enzyme that degrades monoamines in the central nervous system (Seif and De Maeyer, 1999). More specifically, the enzyme catalyzes the oxidative deamination (removal of an amine group) of dopamine and serotonin (Caspi et al., 2002) and has been associated with ADHD and impulsivity (Manuck et al., 2000; Lawson et al., 2003). The rationale behind the selection of each candidate gene is described in Table 1.

**Table 1:** ***Candidate* genes with their biological effects and functional effects**.

Gene	Biological effect	Functional effect
*APOE*	The ε4 haplotype reduces acetylcholine receptor number (Parasuraman et al., 2002) and possibly diminishes synthesis of acetylcholine via impaired regulation of phospholipid and/or fatty acid transport (Poirier, 1996).	APOE has been associated with visuospatial skills in children (Bloss et al., 2010) and appears to have a protective role in cognitive development (Oriá et al., 2010).
*BDNF*	This gene is involved in the serotonergic system (Juckel et al., 2010) as well as nerve growth (Nair and Mishra, 1995).	Related to impulsive-aggressive behaviors in children (Oades et al., 2008)_._
*CHRNA4*	This gene encodes a nicotinic acetylcholine receptor that can bind acetylcholine and open an ion-conducting channel across the plasma membrane. The protein can interact with either nAChR beta-2 or nAChR beta-4 to form a functional receptor (Winterer et al., 2007).	Associated to with ADHD in children (Faraone and Mick, 2010)
*COMT*	G at rs4680 produces valine which is more active in catabolizing dopamine making dopamine less available (Axelrod and Tomchick, 1958).	COMT has been associated with working memory and brain activity during development (Dumontheil et al., 2011).
*DRD4*	Risk alleles lead to fewer dopamine receptors via reduced transcription (Lowe et al., 2004).	There is an association between DRD4, cortical development, and ADHD (Shaw et al., 2007).
*HTR4*	A member of the family of serotonin receptors; the gene product modulates various neurotransmitters (Lambe et al., 2011).	Associated with depression, autism, and ADHD (Faraone and Mick, 2010).
*IGF2*	This gene encodes a member of the insulin family of polypeptide growth factors, which are involved in development (including DA neuron development) and growth (Riikonen et al., 2010). It is an imprinted gene, expressed only from the paternal allele (Kopsida et al., 2011).	Epigenetic changes at this locus are associated with developmental growth disorders such as Beckwith-Wiedemann syndrome (Nativio et al., 2011). Normal intelligence is possible if long term neonatal hypoglycemia is avoided (Obias et al., 1992).
*MAOA*	This gene is on chromosome X. It encodes an enzyme that degrades amine neurotransmitters, such as dopamine and serotonin (Xu et al., 2007).	Abnormal regulation of *MAOA* has been associated with depression, substance abuse, and sexual maturation (Biederman et al., 2008).
*SLC5A7 (aka CHT)*	The presynaptic choline transporter (CHT, SLC5A7) is the major rate-limiting determinant of ACh production in the brain and periphery (Neumann et al., 2005).	Upregulated during tasks that require sustained attention (English et al., 2009).
*SLC6A3(aka DAT1)*	Controls the number of dopamine transporter and therefore less dopamine in the synapse (Giros et al., 1992) and this terminates the dopaminergic signal transmission (Rommelse et al., 2008).	Less dopamine has been associated with greater attentional costs for targets in the left hemifield of children (Bellgrove et al., 2007).
*SNAP25*	This gene product is a presynaptic plasma membrane protein involved in the regulation of neurotransmitter release, including dopamine (Feng et al., 2005).	SNAP25 has been associated with ADHD (Feng et al., 2005).

*Note: Biological effects include changes to the gene that alter the way the protein it codes for is produced (e.g., changes in a synaptic receptor, enzyme, or neurotransmitter). Function effects include associations between this genetic variation and behaviors, including symptoms of disorders associated with attention*.

In particular single nucleotide polymorphisms (SNPs) on each gene were selected based on several attributes including citation in the cognitive literature, minor allele frequency (MAF), block structure, and distance between markers. The markers that we have in the current data set include 39 SNPs and two VNTRs on 11 genes. The genes were selected based on evidence in the literature that the gene was related to attentional deficits or a disorder with an attentional component (see Table 1 for illustrative literature). *COMT, DRD4, IGF2, SLC6A3*, and* SNAP25* are related to the availability of dopamine. *APOE, CHRNA4*, and* SLC5A7* are related to the availability of acetylcholine. *BDNF* and *HTR4* are related to the availability of serotonin while *MAOA* is related to the availability of both dopamine and serotonin. Knowledge about how these markers affect attentional development is sparse (Konrad et al., 2005; Dye and Bavelier, 2010) and so a study with a longitudinal component is likely to be helpful.

### Participants

Participants were recruited after Institutional Review Board (IRB) approval from Rice University (IRB-Human subjects) and the University of Wisconsin-Madison (the Social and Behavioral Sciences IRB). Additional data analysis was performed under IRB approval from Brigham Young University (the IRB for Human Subjects). We invited 345 eligible children who lived in the Madison, Wisconsin area to participate in the computer task study from a population of children who had participated in studies in the Dannemiller lab at the University of Wisconsin-Madison between 1996 and 2001 when they were infants (ages 2–5 months). The infants were full term (±2 weeks) and had normal birthweight (>2500 g). The children were 9–16 years old at the time of our second contact.

Of the 345 invited to participate, 203 participated. All parents gave written informed consent in accordance with the Declaration of Helsinki. Children signed assent forms. The children completed two computer tasks while their parents filled out a questionnaire. The children also provided a saliva sample. The leukocytes (white blood cells) and buccal epithelial cells (from the inner cheek) found in saliva were then used to obtain DNA suitable for genotyping. Two children were excluded for neurological diagnoses and two children were excluded for uncorrected vision diagnoses. After exclusions, we have complete data at two time points for 199 children: one task in infancy and another with the same subjects in childhood.

### Infant Task

**Figure 1:**
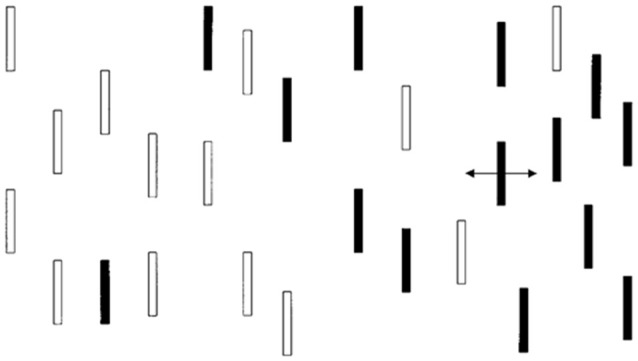
**Monochromatic representation of infant visual display.** The actual presentation had red and green bars.

Infants were shown a display (Dannemiller, 1998) with vertical bars in various configurations (see Figure 1). One of these bars (either on the right or the left side of the screen) oscillated in place horizontally through a visual angle of approximately 0.5–1° at a rate from 1.2 to 2.4 Hz. The rest of the bars remained static. An observer who was unaware of the location of the moving bar made a forced choice of the location of the moving bar (right vs. left) based on the infant’s initial orienting behaviors (e.g., eye movement, facial expression, head or body movements). The judgments were made quickly after the onset of the display with the average judgment taking less than 2 s. Infants who looked selectively at the side of the display with the moving bar were judged (in an age-adjusted manner) to have better reflexive attention since reflexive attention is captured by moving stimuli.

### Child Task

The child task is similar to the infant task except that the bars were monochromatic because contrast and not color were found to be important in the infant task. The static bars are expected to exert potentially distracting effects on eye movements but the moving bar is expected to capture attention most of the time. E-Prime (Psychology Software Tools, Sharpsburg, PA, USA) was used to present stimuli for this task. Participants were digitally recorded with a low-light video camera in a semi-darkened room while performing the task. There were eight practice trials and 16 actual trials. All trial types (display conditions with a moving bar on either the left or right side of fixation and with various placements of distractor bars) were presented in the same order for each participant. All participants were instructed to initially look at a fixation cross (presented as black on a white background) and told that a field of bars would appear (we used a medium gray background with lighter and darker gray bars; see Figure 1). Each child was instructed to look directly at the moving bar as soon as he/she saw it begin to move. The E-Prime code to initiate the onset of movement in one of the bars triggered the initiation of a time stamp imprinted on the digital video file by a FOR-A timer (Tokyo, Japan). The timer is accurate to the nearest 1/100th of a second, but was recorded to the nearest 4/100th of a second during video conversion (required for use of the video editing program described under Preparation of Child Eye Movement Data, below). Two raters coded the 16 trials of each subject for latency (RT) and eye movement direction. Information about eye movement direction was used to later determine PC scores.

### Questionnaire

While the children completed the computer task, the parents completed a questionnaire that included the McArthur Health and Behavior Questionnaire-Parent Version (HBQ-P; Essex et al., 2002; Armstrong et al., 2003). We used a modified form of the HBQ-P that asks parents to use a three-point Likert scale (0–2) to indicate agreement with statements about the behavior of their children. The modified version has been successfully used in a variety of studies (Lemery-Chalfant et al., 2008; Burghy et al., 2012). We examined only the summary scores for *Inattention* as a follow up to trajectory analyses by genotype. The questionnaire also included demographic information (including family income and family size, which we used to approximate SES) and a modified version of the Epworth Sleepiness Scale that is appropriate for children. The Epworth is a simple rating of sleepiness during certain hypothetical, common events (Melendres et al., 2004). In the data analysis, we attempted to determine if either SES or sleepiness (which is associated with not getting enough sleep; Saarenpää-Heikkilä et al., 2000), changed the significance of the model in which *MAOA* predicted changes in RT from infancy to childhood.

### Genotyping Methods

Participants produced a saliva sample of approximately 2 ml in an Oragene-500 kit (DNA Genotek, Kanata, ON, Canada), which was used for genotyping. The Oragene-500 is a plastic test tube with a preservative that is released when the lid is closed. Genotyping for the study was performed using the GoldenGate assay on the BeadXpress system (Illumina, Inc., San Diego, CA, USA). Briefly, the GoldenGate assay involves biotin-labeling of genomic DNA followed by the capture of the labeled DNA onto streptavidin-coated sepharose beads. Streptavidin has a very high affinity and specificity for biotin and thus aids in labeling. Sepharose is a form of agarose that is commonly used in chromatographic separations of biomolecules. An artificial nucleotide-based molecule that contains universal priming sequences on either end and is complimentary to the target DNA sequence of interest is then created, amplified and hybridized to holographically-labeled silica bars that form arrays with up to 30-fold redundancy of each target to be interrogated. Once the array has been visualized with the BeadXpress reader, wavelength and intensity values of the fluorescence are used to determine the genotype. A custom Laboratory Information Management System is used to track both samples and laboratory throughput. Allele detection and genotype calling were performed using GenomeStudio software v2011.1 (Illumina, Inc.).

### Statistical Procedures

#### Preparation of Infant Scores

In the infant data set there is a PC score for each infant, which indicates the percentage of trials that a single observer determined that the infant oriented to the side with the moving bar. Each infant also has an average latency to orient to the moving bar that represents the RT of the observer to make a decision concerning orientation. PC scores are proportions and therefore received a logit transform. Infant scores were corrected for gestational age and study conditions by saving standardized residual scores after regressing the variables on the transformed PC and RT values. These two residual measures were used separately in analyses.

#### Preparation of Child Eye Movement Data

Because the child eye movement data was coded by multiple video coders, we used inter-rater reliability (IRR) analyses to determine if it was reasonable to aggregate data from two raters. We used a video editing software (Pinnacle, Corel Corporation, Mountain View, CA, USA) to analyze the latency and direction of the eye movements. Video coders worked independently and each video was coded twice. Video coders were trained to code the very first eye movement for direction and latency even if the child self-corrected for direction after this first look. Focusing on the first eye movement captures distractibility in the children and provides variability in the data. Raters advanced each trial frame-by-frame beginning with the presentation of the stimulus until the first eye movement was noted. Latency was recorded based on the time stamp at this initial eye movement. Training continued until acceptable IRR (set at 0.80). After averaging raters’ data codes, the eye movement direction was compared to the stimulus presentation side and coded as correct or incorrect.

Agreement on eye direction was very high. Discrepancies (where one rater coded left and one coded right) occurred in 3% of trials. An additional 3% could not be coded and are considered missing data. Overall IRR across all dyads was 0.95 for RT and 0.93 for eye movement direction. Therefore, the two ratings were averaged. For each subject, PC was calculated and RT for *correct* trials were aggregated (averaged) per person. After averaging across raters, all the RTs for left targets and all the RTs for right targets are again averaged within each subject and these are not meaningfully skewed (0.96), making the observations normal enough to use in statistical procedures that require normal data.

Raw PC and RT scores were adjusted for age and (in the case of infant scores) study number by saving standardized residual scores after regressing the variables on the transformed PC and RT values. Residual PC and RT scores were used separately in analyses. We examined trajectory using mixed (multilevel) modeling in SPSS 22 (IBM, 2013) using the MIXED command. Multilevel modeling accounts for the nested nature of the data (i.e., that trials can be attributed to individuals). We used this method because the intraclass correlation indicated that 75% of the variance came from the individual-level data (level 2) and we needed to take the nested nature of the data into account when analyzing time point data (level 1). Each gene had two to seven genetic markers (SNPs or VNTRs) that were included as predictors because they were not closely linked with other available markers on that gene. We used separate analyses per gene because a longitudinal study with only two time points has difficulty with more complex models with more predictors and it was important that we be able to test interactions with time point to answer our research questions. We performed 11 sets of analyses using a backwards design similar to backwards regression. Each set of analyses included an empty model (with no predictors), a full model (with all predictors and interactions as described below), and a reduced model with no interactions. Final models were determined by comparison to an empty model with no predictors and to the full model with the following predictors: all markers on a given gene, time point, lag time, and (in the case of *MAOA*, which is on the X chromosome) sex. The only interactions that were included were between genetic markers and time point and (in the case of *MAOA*) between genetic marker, time point and sex. Essentially, the multilevel model is fitting a line for each person through the transformed scores of each person and then the interaction between time point and genotype indicates if the trajectory is affected by genotype.

We entered each set of markers and each markers interaction with the time component (time 1 for infancy and time 2 for childhood) and the lag time between infant testing and child testing as predictors of (in turn) residual RT and residual PC. The interaction between time point and genetic marker indicates different trajectories depending on genotype. The output of interest when examining outcome trajectories over the course of a task is the interaction between the genetic marker and time point. We are looking for time point by marker interactions by modeling transformed RT and PC separately as the dependent variables (the outcome in the multilevel model). We used the lag time as a covariate to account for variability between participants’ two age point differences. A participant in this study was tested once as an infant and once as a child, so we calculated lag time by subtracting age corrected for gestational age (age at the first time they were tested), from the age of the child (age at the second time they were tested). We kept lag time in units of days, as days in the life of a small child can play a major role in physical and cognitive maturity. Then we used the lag time as a covariate to take into account the role of time and hence increases the potential to view with more clarity the influence that genes play in reflexive attention over time.

## Results

### Hardy-Weinberg Equilibrium

We performed chi-square tests to determine if alleles and genotypes were present in the expected proportions according to the Hardy-Weinberg principle (Hardy, 1908). Non-significant results indicate the absence of increased evolutionary influence such as genetic drift, mutation, or biases in mate selection. These tests were performed using the whole sample, prior to excluding subjects. All SNPs were in Hardy-Weinberg equilibrium (all *P*s > 0.39) indicating no cause for concern.

### Behavioral Results

Fifty-one percent (*n* = 102) of the sample was male. Infants ranged in age from 1.51 to 5.39 months (*M* = 3.63) and had raw PC scores from 42 to 98% (*M* = 0.72) and raw RT from 1080.52 to 3180.00 ms (*M* = 1804.92 ms). The children ranged in age from 10.58 to 16.55 years (*M* = 12.93) and had raw PC scores from 44 to 100% (*M* = 0.84) and raw RT from 274.52 to 500.71 ms (*M* = 361.74). There was a significant correlation between infant and child standardized residual RT scores (*r* = 0.29, *p* < 0.001). This indicates that the infant and the child constructs for RT are similar. However, infant and child PC scores were not correlated (*r* = 0.05, *p* = 0.48). Note that prior to creating the residual PC scores, we adjusted raw PC scores downward so that perfect (100%) scores were converted to 99% scores. This allowed us to take the logit transform and to perform linear-based regression. Ages by sex and residual PC and RT scores are shown in Table 2.

**Table 2:** **Dependent variables after controlling for age by time point and sex**.

		AGE (months)		Residual PC		Residual RT	
		Mean	SD	Mean	SD	Mean	SD
Time 1 (Infancy)	Male	3.61	0.83	0.11	0.97	0.06	1.21
	Female	3.64	0.73	0.08	1.12	−0.05	1.07
	Combined	3.62	0.79	0.10	1.05	0.00	1.14
Time 2 (Childhood)	Male	154.11	21.69	−0.06	1.01	−0.10	0.63
	Female	155.06	20.23	0.11	1.00	−0.06	0.63
	Combined	154.57	20.95	0.02	1.00	−0.08	0.63

*Abbreviations: PC, percent correct; RT, reaction time*.

### Multilevel Analysis

All genetic models with interactions with time point were better than models without interactions with time point (*P*s < 0.001). All models with interactions were significant after correcting for multiple comparisons using a sequential Bonferroni procedure (Benjamini and Hochberg, 1995). Adjusted alpha levels ranged from 0.005 to 0.05. We examined Schwarz’s Bayesian Information Criterion (*BIC*) to compare models using chi-square statistics. Smaller *BIC* values are better and the chi-square value indicates if a model is significantly better. Once the best model was determined, individual predictors and interactions were examined for significance using the fixed effects output. Only the *MAOA* model had a significant interaction of interest that included time point.

As can be seen in Table 3, the *MAOA* model with interactions between genetic markers, time point, and sex had the smallest *BIC* at 705.38 and was significantly better than the empty model (*BIC* = 1015.65), ${\text{χ}}_{\left(20,N=398\right)}^{2}$ = 246.55, *p* < 0.001. This *p*-value (5.66 × 10^41^) is substantially less than the family-wise alpha of 0.004. The model with fewer interactions (*BIC* = 705.83) was a little worse than this model. Neither the model with SES (*BIC* = 712.76) nor the model with sleepiness (*BIC* = 706.76) were better than the model with interactions between the *MAOA* markers, time point and sex.

**Table 3:** **Parameter estimates from models predicting residual RT with *MAOA***.

		Empty		Full		Simplified		Add Sleep		Add SES	
	Parameter	Mean	SE	Mean	SE	Mean	SE	Mean	SE	Mean	SE
Fixed Effects											
Intercept		−0.06	0.04	−6.36**	0.60	−6.36**	0.60	−6.23**	0.59	−10.16**	0.75
Level 1 (time point)										
	Infant			4.78**	1.53	4.76**	1.53	4.56**	1.53	5.72**	0.98
	Child			0^a^	0.00	0^a^	0.00	0^b^	0.00	0^b^	0.00
Level 2 (individual)										
	rs1137070 (0 risk)			0.27	0.57	0.27	0.56	0.35	0.56	3.91**	0.50
	(1 risk)			0.05	0.31	0.05	0.31	0.08	0.31	1.87**	0.30
	(2 risk)			0^a^	0.00	0^a^	0.00	0^b^	0.00	0^b^	0.00
	rs12843268 (0 risk)			2.21**	0.34	2.21**	0.34	2.17**	0.33	4.01**	0.44
	(1 risk)			2.06**	0.21	2.06**	0.20	2.00**	0.20	2.04**	0.20
	(2 risk)			0^a^	0.00	0^a^	0.00	0^b^	0.00	0^b^	0.00
	rs909525 (0 risk)			0.13	0.18	0.13	0.16	0.10	0.18	0.16	0.18
	(1 risk)			0.13	0.14	0.13	0.14	0.11	0.14	0.16	0.14
	(2 risk)			0^a^	0.00	0^a^	0.00	0^b^	0.00	0^b^	0.00
	lag time			0.00**	0.00	0.00**	0.00	0.00**	0.00	0.00**	0.00
	sex			0.22	0.32	0.22	0.27	0.25	0.27	2.05**	0.25
	sleepiness									0.02**	0.01
	SES									0.00*	0.00
	time 1 * rs1137070 (0 risk)			2.01	1.47	1.57	1.42	2.00	1.46	1.12	0.91
	(1 risk)			0.58	0.70	0.54	0.70	0.60	0.70	0.50	0.69
	(2 risk)			4.20	1.56	0^a^	0.00	0^a^	0.00	0^b^	0.00
	time 2 * rs1137070 (0 risk)			0^a^	0.00	0^a^	0.00	0^a^	0.00	0^b^	0.00
	(1 risk)			0^a^	0.00	0^a^	0.00	0^a^	0.00	0^b^	0.00
	(2 risk)			0^a^	0.00	0^a^	0.00	0^a^	0.00	0^b^	0.00
	time 1 * rs12843268 (0 risk)			−1.07	1.48	−1.06	1.48	−1.02	1.47	−1.89*	0.89
	(1 risk)			−1.88*	0.86	−1.84*	0.86	−1.80**	0.86	−1.89*	0.85
	(2 risk)			0^a^	0.00	0^a^	0.00	0^a^	0.00	0^a^	0.00
	time 2 * rs12843268 (0 risk)			0^a^	0.00	0^a^	0.00	0^a^	0.00	0^a^	0.00
	(1 risk)			0^a^	0.00	0^a^	0.00	0^a^	0.00	0^a^	0.00
	(2 risk)			0^a^	0.00	0^a^	0.00	0^a^	0.00	0^a^	0.00
	time 1 * rs909525 (0 risk)			−0.94	0.79	−0.49	0.70	−0.95	0.79	−0.99	0.78
	(1 risk)			−0.32	0.62	−0.30	0.62	−0.33	0.62	−0.34	0.61
	(2 risk)			0^a^	0.00	0^a^	0.00	0^a^	0.00	0^a^	0.00
	time 2 * rs909525 (0 risk)			0^a^	0.00	0^a^	0.00	0^a^	0.00	0^a^	0.00
	(1 risk)			0^a^	0.00	0^a^	0.00	0^a^	0.00	0^a^	0.00
	(2 risk)			0^a^	0.00	0^a^	0.00	0^a^	0.00	0^a^	0.00
	time 1 * rs1137070 (0 risk) * sex			−1.00	1.08	−0.26	0.87	−1.02	1.07	−2.84**	1.05
	(1 risk)			0.80	1.20	0.82	1.20	0.77	1.20	0^a^	0.00
	(2 risk)			0^a^	0.00	0^a^	0.00	0^a^	0.00	0^a^	0.00
	time 2 * rs1137070 (0 risk) * sex			1.83**	0.38	1.83**	0.34	1.72**	0.38	0^a^	0.00
	(1 risk)			0^a^	0.00	0^a^	0.00	0^a^	0.00	0^a^	0.00
	(2 risk)			0^a^	0.00	0^a^	0.00	0^a^	0.00	0^a^	0.00
	time 1 * rs12843268 (0 risk) * sex			0^a^	0.00	0^a^	0.00	0^a^	0.00	0^a^	0.00
	(1 risk)			0^a^	0.00	0^a^	0.00	0^a^	0.00	0^a^	0.00
	(2 risk)			0^a^	0.00	0^a^	0.00	0^a^	0.00	0^a^	0.00
	time 2 * rs12843268 (0 risk) * sex			0^a^	0.00	0^a^	0.00	0^a^	0.00	0^a^	0.00
	(1 risk)			0^a^	0.00	0^a^	0.00	0^a^	0.00	0^a^	0.00
	(2 risk)			0^a^	0.00	0^a^	0.00	0^a^	0.00	0^a^	0.00
	time 1 * rs909525 (0 risk) * sex			0.77	0.65	−0.95	0.79	0.79	0.65	0.80	0.64
	(1 risk)			0^a^	0.00	−0.33	0.62	0^a^	0.00	0^a^	0.00
	(2 risk)			0^a^	0.00			0^a^	0.00	0^a^	0.00
	time 2 * rs909525 (0 risk) * sex			0.00	0.15			0.03	0.15	−0.01	0.15
	(1 risk)			0^a^	0.00			0^a^	0.00	0^a^	0.00
	(2 risk)			0^a^	0.00			0^a^	0.00	0^a^	0.00
Repeated	Time point 1	1.30	0.13	1.33	0.14	1.34	0.14	1.32	0.14	1.28	0.13
Measures	Time point 2	0.40	0.04	0.72	0.01	0.07	0.01	0.07	0.01	0.07	0.01
Num. of Parameters		3.00		23		21		24		22	
Schwarz’s BIC		1015.65		705.38		705.83		706.76		712.76	

**Significant at p < 0.01. *Significant at p < 0.05. Significant values indicate that the estimate is different from zero. We report results under tests of fixed effects for gene by trial interactions to determine if trajectories differ by genotype. ^a^This parameter has been set to zero. Note that this Table shows model parameters when testing residual RTs.

**Figure 2:**
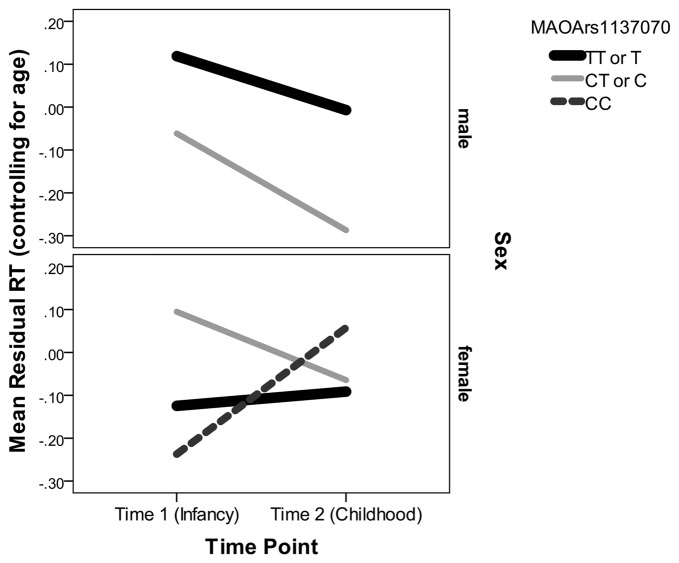
**Mean residual reaction time (RT; controlling for age) represents the subject’s position in the distribution of infant or child scores at each time point.** Because Monoamine oxidase A (MAOA) is on the X chromosome, it is only possible for boys to have one of two genotypes. Boys with either genotype decrease (improve) in their relative RT between infancy and childhood. Girls with the CT genotype decrease in their relative RT but girls with the CC genotype increase in their relative RT.

Examining the best model, we see that an interaction of interest is significant, *F*_(2,188.50)_ = 12.50, *p* < 0.001. Multilevel modeling is regression based, and so this indicates that even after controlling for all other predictors and interactions in the model, the trajectory of residual RT varies according to sex and genotype on the rs1137070 SNP of *MAOA* (see Figure 2). This information is also presented in tabular form in Table 4, which includes the genotype counts by sex.

**Table 4:** **Means and standard deviations of RT and residual RT scores by time point and sex**.

				Raw RT		Residual RT	
			Count	Mean	SD	Mean	SD
Infancy	Male	T	67	1840.75	418.98	0.12	1.33
		C	35	1784.86	293.76	−0.06	0.93
	Female	TT	48	1763.96	303.61	−0.12	0.97
		CT	38	1834.21	391.54	0.09	1.25
		CC	11	1728.18	259.92	−0.24	0.82
Childhood	Male	T	67	364.10	45.20	−0.01	0.66
		C	35	352.60	27.89	−0.29	0.54
	Female	TT	48	364.52	39.55	−0.09	0.60
		CT	38	365.49	37.97	−0.06	0.66
		CC	11	351.30	29.73	0.06	0.72

*Note. Age was controlled by residualizing RT within time point and saving the standardized scores. Residual RT can be interpreted as a Z-score*.

There were also main effects for lag time (*F*_(1,188)_ = 860.64, *p* < 0.001), sex (*F*_(1,209.61)_ = 4.98, *p* = 0.03), and time point (*F*_(1,374.08)_ = 488.92, *p* < 0.001)—but not for the other *MAOA* SNPs (*P*s > 0.21) or their interactions with time point and/or sex (*P*s > 0.22). In the interaction with rs1137070, boys in either the zero (T genotype) or one (C genotype) risk-allele group improved their position in the distribution of boys and girls (because the residual RT scores can be interpreted as *z*-scores controlling for age with lower RTs indicating increased speed). Girls with zero risk alleles (the TT genotype) approximately retained their position in the distribution. Those with one risk allele (the TC genotype) improved their position in the distribution, and those with two-risk alleles (the CC genotype group) sharply increased (worsened) their position in the distribution. It is interesting that girls with TT or CT genotypes start out differently, but converge on similar RTs. This finding is unusual; however, it possibly indicates heterozygote disadvantage (aka underdominance). This phenomenon concerns situations where homozygotes are associated with a more beneficial phenotype but heterozygotes with a less desirable phenotype. This is the reverse situation to that of sickle cell anemia, in which heterozygotes are at an advantage. Two possible human examples of heterozygote disadvantage in humans are Rh factor incompatibility, which can lead to hemolytic anemia in a fetus (Cavalli-Sforza and Bodmer, 1971), and polymorphisms in the *MTHFR* gene, which influence embryo implantation (Enciso et al., 2016). While less fit heterozygotes may eventually become rarer, this is likely to take at least 100 generations (O’Fallon and Adler, 2006; Peischl and Bürger, 2008) and takes longer under certain conditions. For example, the stable heterozygote disadvantage is more likely when the heterozygote state has a weak influence on survival, the same allele leads to both non-advantageous and advantageous traits (i.e., is pleiotropic), and it is linked to other loci (Wilson and Turelli, 1986; Eppstein et al., 2009; Lawson et al., 2011). This is interesting because *MAOA* is a gene with high linkage between loci. In addition, the change we witnessed in the distributional position of RT does not appear to have strong influence on survivability, and (due to the action of the MAOA enzyme) there may be some biological advantage to having a slightly more dopamine available in some situations (Cools and D’Esposito, 2011). Nevertheless, this explanation should be treated with caution until this study is replicated and shows a pattern suggesting heterozygote disadvantage for RT distributional position on a similar attention task.

As a follow up analysis, we examined if the girls in the CC genotype group had more symptoms of inattention as measured by the HBQ-P. There were more inattention symptoms (*F*_(2,97)_ = 3.29, *p* = 0.04). This indicates that girls in the CC genotype group are being identified by parents as having trouble with attention and are declining in task performance between infancy and childhood relative to their peers.

## Discussion

### Summary and Implications of Results

We found evidence that the SNP rs1137070 on *MAOA* predicted poorer developmental course in RT such that girls with the CC genotype show an increase in (slowing of) RT between two time points (infancy and childhood) on a reflexive attention task. In contrast, the analogous genotype on *MAOA* was not associated with slowing RT across development in boys. The effect of genotype on RT remained even when controlling for SES and sleepiness. We also note that our findings are unlikely to be related to IQ because the children’s academic reading scores (which are frequently used to approximate IQ; Manolakes and Sheldon, 1955; Kaufman et al., 2012) were not associated with their positional change in the distribution of scores from infancy to childhood. We also note that a trend for this significant interaction (*p* = 0.12) remained when we removed likely prepubertal children (males younger than 11.66 years; all females had likely already begun puberty [i.e., were older than 9.86 years]; Lee and Styne, 2013). The drop in significance probably represents a loss of power with a smaller sample size. Thus, we do not believe that the prepubertal children are driving significance.

Growing evidence suggests that the *MAOA* genotype may play a role in attention and cognition (Guinmarães et al., 2009; Wargelius et al., 2012; Ernst et al., 2013; Nymberg et al., 2013; Piton et al., 2014). However, to gain confidence in these findings, the behavioral phenotype needs to make sense with biological actions of *MAOA*. The *MAOA* gene encodes an enzyme that degrades amines such as dopamine, norepinephrine and serotonin (Garrett and Soares-da-Silva, 1990). The rs1137070 SNP (formerly rs1801291 and aka codon 1460) is in exon 14. T alleles have been found to be associated with increased MAOA activity (Hotamisligil and Breakefield, 1991), and may therefore regulate gene expression (Zhang et al., 2010). Increased levels of MAOA (the enzyme) lead to decreased levels of dopamine. Also, the serotonin system inhibits the firing of the dopaminergic system at the midbrain (Jacobs and Azmitia, 1992), therefore decreasing the likelihood of activating the frontal dopamine system.

We gain confidence in our finding because it is consistent with previous longitudinal studies showing an association between *MAOA* and negative outcomes that could have a cognitive component (Edwards et al., 2010; Enoch et al., 2010; Belsky and Beaver, 2011; Daw and Guo, 2011; Fergusson et al., 2011; Lee, 2011; Hill et al., 2013; Pickles et al., 2013; Priess-Groben and Hyde, 2013; Haberstick et al., 2014; Whelan et al., 2014). Such findings establish heterotypic continuity (Putnam et al., 2008; Miller et al., 2009; Lavigne et al., 2014) between the infant reflexive attention task and later child behavior.

To the best of our knowledge, however, there has only been one other longitudinal molecular genetic studies of attention children that included *MAOA* (Zohsel et al., 2015). This study found that the *MAOA* VNTR plays a role in determining continuity of parent-rated attention problems during adolescence. Attention problems during early adolescence (at 11 years old) were found to be strong predictors of attention problems in middle adolescence (at 15 years old). However, stability of attention in carriers of the low-activity variant (MAOA-L) was higher than in carriers of the high-activity variant (MAOA-H).

Our finding that *MAOA* (but not the other genes tested) is associated with RT changes between infancy and childhood suggests that *MAOA* has a particular role in the development of attention. This role might involve *MAOA*’s function in the pre-synaptic membrane release of dopamine (Yu et al., 2005). In addition, the role could involve *MAOA*’s involvement in degrading monoamines such as dopamine in the central nervous system (Seif and De Maeyer, 1999). These roles could change developmentally over the age range we examined. For example, if dopamine drives axon guidance, cell mobility, and synapse formation early in life, but shifts to synapse function and messaging later in childhood (Gilman et al., 2012; Douet et al., 2014), then the change in roles could lead to individual differences in attentional trajectory, which might be adequate at one stage of development but not at another.

Examining the positional changes in the distribution is important because of the large developmental differences between infancy and childhood. For example, note in Table 4 that the absolute magnitude of the differences between infancy and childhood is large in terms of raw RT (the range is from 1377 to 1467 ms, depending on genotype groups compared). This is expected because children are much faster at responding than infants (or, in this case, an observer who is judging infant responses). The large difference is one reason we used standardized residual values as the dependent variable. However, the difference in improvement (infant RT—child RT) is much smaller (varying from 23 to 90 ms depending on genotype groups compared). Note that these values are in the range of noticeable differences (Shackleton et al., 2003).

Also in Table 4, it is apparent that the CC genotype group is small (11 girls). This might suggest one possible reason for the group differences in standardized residual RT. However, small group size generally increases variability and decreases the ability to detect a significant difference. In addition, notice that the CC group is the least variable of the three genotype groups for girls.

Our finding that the SNP rs1137070 on *MAOA* predicted poorer developmental course in girls with the CC genotype does not detract from the relevance for the other genes in association at a single time point with attention or cognition. Several of the genes we tested, including *BDNF*, *CHRNA4*, *COMT*, *DRD4*, *HTR4*, *SLC6A3*, *SLC5A7*, and *SNAP25*, have been associated with ADHD. In our study, all 11 genes were associated with task performance in terms of RT. They simply were not associated with trajectory, which is the interaction between time point and genetic marker that we set out to test. It appears, therefore, that these genes do not influence the development of change in position in a distribution of RT scores after controlling for age. Another possibility is that these genes do influence RT score position, but that our sample size was not sufficient to detect the size of the effect. If this is true, it is still important to note that the effect was stronger with *MAOA*.

In addition to explicating the mechanisms by which symptoms of inattention emerge in a general population of children, there are some intriguing clinical implications of our findings. First, if young girls with the CC genotype on rs1137070 can be identified early, they can be monitored in a new longitudinal study to determine more precisely the course of their development and concomitant risk factors such as those involved with various life experiences at home and school. This study should involve many time points and verify the genes involved in predicting risk and the eventual development of inattentive symptoms. Although it is unclear how reflexive attention and sustained attention are related (Hikosaka et al., 1993; Suzuki and Cavanagh, 1997; Barry et al., 2001; Berger et al., 2005; Andrade et al., 2009; Henderickx et al., 2010; Carrasco, 2011; Macaluso and Doricchi, 2013; Underbjerg et al., 2013; Dye and Hauser, 2014; Anderson, 2015), my previous work has indicated that reflexive attention is related to day-to-day attentional performance (Lundwall, 2013). Second, in conjunction with the above, several comparison intervention programs could be targeted earlier to determine which are most effective. An intervention study would also move our knowledge from observational to causal. Clinical resources such as the type and intensity of intervention ideally need to be tailored according to individual differences. Predictors, both clinical and developmental, could shape resources to become more effective. In addition, identifying genetic and environmental risk factors that influence the developmental course of inattentive symptoms is important because it identifies the mechanisms that lead to poorer clinical outcomes.

Information on the development of attentional pathways and other cognitive development comes from studies of children with ADHD. Marx et al. (2010) tested the domains of working memory (prefrontal cortex) interference control (frontocortical circuits), time perceptions (frontostriatal-cerebellar circuits) and delay aversion (striatal-limbic circuits) in male children, adolescents, and young adults. Although the study was cross-sectional, the authors conclude that cognitive deficits in the domains tested tended to persist across the lifespan. Others have noted that cognitive symptoms of ADHD tend to persist even when the behavioral symptoms resolve (Biederman, 1998; Biederman et al., 2009) and that clinical improvements tend to parallel brain volume normalization (Giedd and Rapoport, 2010). Because ADHD symptoms exist on a continuum in the general population, this suggests the possibility of similar patterns to those described above in children from the general population, which seems to be confirmed by the persistence of the influence of *MAOA* on attention in a general population of children (Zohsel et al., 2015). There were no studies extending these findings into young adulthood, and this an important goal of future research.

### Study Limitations and Future Directions

Our study has several limitations. For example, three time points would provide more certainty in the time trends we found. We do note that multilevel modeling of two time points has been used successfully by other researchers in other fields (Bere et al., 2011; Hearst et al., 2012; Normand et al., 2012; Murray et al., 2013; Nicholson et al., 2014). However, future replication attempts should include more time points and it would be especially helpful to continue this study into young adulthood. In addition, both time points and sample size matter in determining the complexity of the models that can be tested with the data. The sample size (*N* = 199) was somewhat small once genetic subgroups are formed. Scherbaum and Ferreter (2009) discusses several factors related to power in multilevel modeling, which suggest that a minimum of 30 individuals each with 30 time points be used for power to detect cross-level interactions (the kind of interactions we are testing), but indicates that a larger number of individuals can compensate for a smaller number of time points. Because we found an association, this suggests sufficient power. Nevertheless, additional subjects would increase confidence in the results of any replication.

Another limitation concerns the possibility of measurement variance. Measurement invariance is sometimes an issue because the construct of reflexive attention has different meanings given the rapid physical, cognitive, and socio-emotional development between infancy and childhood (Glück and Indurkhya, 2001; Bontempo et al., 2012). For example, the development of language means we can give instructions to children that infants cannot follow. In this case, the measurement variance (or construct shift) refers to the lack of similarity between PC measures of attention at the two ages. Infant and child RT scores were correlated. This is, admittedly, a very basic look at establishing that the constructs were substantively similar. A more complete analysis would involve multiple measures of the construct of attention at each age and structural equation modeling. This was not possible with the available data. Tracking construct shift at multiple ages would allow for more through interpretation of any shifts that do occur, such as those that seem likely for the PC scores. Another possibility is to use a few different age-appropriate tasks at each time point in an overlapping design and use structural equation modeling to track constructs through time. This method was successfully used by Petersen et al. (2015). Thus, there are ways to investigate the meaning of the constructs across development in future studies.

It is also important for future studies to expand on our Caucasian sample. This was intentional to avoid issues with population stratification, which is simply confounding between genotype and phenotype that can occur when ancestral heritage influences both. However, using only Caucasians does limit generalizability to other groups. The best way to handle this is by replicating this study in each of the other ethnic groups available in a researcher’s region.

Future replication efforts that identify genetic and other risk factors that influence the developmental course of attention is important for several reasons. First, it is important to identify the mechanisms that lead to poorer clinical outcomes. Second, clinical resources such as intensity of followup ideally need to be tailored according to need. Predictors, both clinical and etiological, could thus help target resources more effectively. The identification of non-genetic factors and how they may interact with genetic variants in influencing the developmental course is also an important area for future research, although larger longitudinal samples will be needed when testing for Gene × Environment interactions.

## Conclusion

In conclusion, we find evidence that *MAOA* is associated with different developmental patterns from infancy to childhood (over a rather long time span of 15 years). Importantly, we also find evidence that the effect of this gene on attention can vary as a function of sex. Identifying how specific risks are associated with identified genes will be necessary to advance our ability to design more effective prevention and intervention programs for individuals at risk. These analyses underscore the importance of studying genetic effects across development and of identifying factors that influence risk.

## Author Contributions

RAL designed the study, collected the data, supervised the statistical analysis, and wrote the manuscript. CGR performed the statistical analysis and wrote portions of the manuscript. Both authors reviewed and approved the final manuscript.

## Funding

The research was supported in part by grants from the Social Sciences Research Institute at Rice University (Dissertation Improvement Grant to RAL and Seed Money Grant to JLD) and by the Lynette S. Autrey Fund (to JLD). Infrastructure support was provided by the Waisman Center (University of Wisconsin-Madison) via a core grant from National Institute of Child Health and Human Development (NICHD; P30 HD03352).

## Conflict of Interest Statement

The authors declare that the research was conducted in the absence of any commercial or financial relationships that could be construed as a potential conflict of interest.

## Abbreviation

SC: spatial cueing.

## References

[B1] Abed M. (2014). The diagnosis, intervention and treatment of ADHD. A critical review. J. Am. Sci. 10, 114–129. 10.7537/marsjas100814.16

[B2] Anderson B. A. (2015). The attention habit: how reward learning shapes attentional selection. Ann. N Y Acad. Sci. 1369, 24–39. 10.1111/nyas.12957 26595376

[B3] Andrade B. F. Brodeur D. A. Waschbusch D. A. Stewart S. H. McGee R. (2009). Selective and sustained attention as predictors of social problems in children with typical and disordered attention abilities. J. Atten. Disord. 12, 341–352. 10.1177/1087054708320440 18596299

[B4] Annaz D. Van Herwegen J. Thomas M. Fishman R. Karmiloff-Smith A. Rundblad G. (2009). Comprehension of metaphor and metonymy in children with Williams syndrome. Int. J. Lang. Commun. Disord. 44, 962–978. 10.1080/13682820802525005 19874091

[B5] Armstrong J. M. Goldstein L. H. The MacArthur Working Group on Outcome Assessment (2003). “Manual for the MacArthur health and behavior questionnaire (HBQ 1.0),” in MacArthur Foundation Research Network on Psychopathology and Development, ed. Kupfer D. J. (Pittsburgh, PA: University of Pittsburgh).

[B6] Axelrod J. Tomchick R. (1958). Enzymatic O-methylation of epinephrine and other catechols. J. Biol. Chem. 233, 702–705. 13575440

[B7] Bar-Haim Y. Morag I. Glickman S. (2011). Training anxious children to disengage attention from threat: a randomized controlled trial. J. Child Psychol. Psychiatry 52, 861–869. 10.1111/j.1469-7610.2011.02368.x 21250993

[B8] Barry T. D. Klinger L. G. Lyman R. D. Bush D. Hawkins L. (2001). Visual selective attention versus sustained attention in boys with attention-deficit/hyperactivity disorder. J. Atten. Disord. 4, 193–202. 10.1177/108705470100400401

[B9] Bellgrove M. A. Chambers C. D. Johnson K. A. Daibhis A. Daly M. Hawi Z.. (2007). Dopaminergic genotype biases spatial attention in healthy children. Mol. Psychiatry 12, 786–792. 10.1038/sj.mp.4002022 17549062

[B500] Belsky J. Beaver K. M. (2011). Cumulative-genetic plasticity, parenting and adolescent self-regulation. J. Child Psychol. Psychiatry 52, 619–626. 10.1111/j.1469-7610.2010.02327.x 21039487 PMC4357655

[B10] Benjamini Y. Hochberg Y. (1995). Controlling the false discovery rate: a practical and powerful approach to multiple testing. J. R. Stat. Soc. Series B Stat. Methodol. 57, 289–300.

[B11] Bere E. Oenema A. Prins R. G. Seiler S. Brug J. (2011). Longitudinal associations between cycling to school and weight status. Int. J. Pediatr. Obes. 6, 182–187. 10.3109/17477166.2011.583656 21644849

[B12] Berger A. Henik A. Rafal R. (2005). Competition between endogenous and exogenous orienting of visual attention. J. Exp. Psychol. Gen. 134, 207–221. 10.1037/0096-3445.134.2.207 15869346

[B13] Bidwell L. C. Willcutt E. G. McQueen M. B. DeFries J. C. Olson R. K. Smith S. D.. (2011). A family based association study of DRD4, DAT1 and 5HTT and continuous traits of attention-deficit hyperactivity disorder. Behav. Genet. 41, 165–174. 10.1007/s10519-010-9437-y 21207241 PMC3674022

[B14] Biederman J. (1998). Attention-deficit/hyperactivity disorder: a life-span perspective. J. Clin. Psychiatry 59, 4–16. 9680048

[B15] Biederman J. Kim J. W. Doyle A. E. Mick E. Fagerness J. Smoller J. W.. (2008). Sexually dimorphic effects of four genes (COMT, SLC6A2, MAOA, SLC6A4) in genetic associations of ADHD: a preliminary study. Am. J. Med. Genet. B Neuropsychiatr. Genet. 147B, 1511–1518. 10.1002/ajmg.b.30874 18937309 PMC2587524

[B16] Biederman J. Petty C. R. Ball S. W. Fried R. Doyle A. E. Cohen D.. (2009). Are cognitive deficits in attention deficit/hyperactivity disorder related to the course of the disorder? A prospective controlled follow-up study of grown up boys with persistent and remitting course. Psychiatry Res. 170, 177–182. 10.1016/j.psychres.2008.09.010 19900713 PMC2787767

[B17] Binder D. K. Scharfman H. E. (2004). Brain-derived neurotrophic factor. Growth Factors 22, 123–131. 10.1080/08977190410001723308 15518235 PMC2504526

[B18] Bloss C. S. Delis D. C. Salmon D. P. Bondi M. W. (2010). APOE genotype is associated with left-handedness and visuospatial skills in children. Neurobiol. Aging 31, 787–795. 10.1016/j.neurobiolaging.2008.05.021 18606479 PMC2865468

[B19] Bontempo D. E. Grouzet F. E. Hofer S. M. (2012). “Measurement issues in the analysis of within-person change,” in Longitudinal Data Analysis: A Practical Guide for Researchers in Aging, Health and Social Sciences, eds Newsome J. T. Jones R. N. Hofer S. M. (New York, NY: Routledge), 97–142.

[B20] Borenstein A. R. Copenhaver C. I. Mortimer J. A. (2006). Early-life risk factors for Alzheimer disease. Alzheimer Dis. Assoc. Disord. 20, 63–72. 10.1097/01.wad.0000201854.62116.d7 16493239

[B21] Bornstein M. H. Sigman M. D. (1986). Continuity in mental development from infancy. Child Dev. 57, 251–274. 10.2307/1130581 3956312

[B23] Brookes K. Xu X. Chen W. Zhou K. Neale B. Lowe N.. (2006). The analysis of 51 genes in DSM-IV combined type attention deficit hyperactivity disorder: association signals in DRD4, DAT1 and 16 other genes. Mol. Psychiatry 11, 934–953. 10.1038/sj.mp.4001869 16894395

[B24] Burghy C. A. Stodola D. E. Ruttle P. L. Molloy E. K. Armstrong J. M. Oler J. A.. (2012). Developmental pathways to amygdala-prefrontal function and internalizing symptoms in adolescence. Nat. Neurosci. 15, 1736–1741. 10.1038/nn.3257 23143517 PMC3509229

[B25] Camarata S. (2014). Early identification and early intervention in autism spectrum disorders: accurate and effective? Int. J. Speech Lang. Pathol. 16, 1–10. 10.3109/17549507.2013.858773 24410017

[B26] Carrasco M. (2011). Visual attention: the past 25 years. Vision Res. 51, 1484–1525. 10.1016/j.visres.2011.04.012 21549742 PMC3390154

[B27] Caspi A. McClay J. L. Moffitt T. E. Mill J. Martin J. Craig I. W.. (2002). Role of genotype in the cycle of violence in maltreated children. Science 297, 851–854. 10.1126/science.1072290 12161658

[B28] Cavalli-Sforza L. Bodmer W. (1971). The Genetics of Human Populations. San Francisco, CA: WH Freeman.

[B29] Colombo J. (1993). Infant Cognition: Predicting Later Intellectual Functioning. Thousand Oaks, CA: Sage Publications Inc.

[B30] Cools R. D’Esposito M. (2011). Inverted-U-shaped dopamine actions on human working memory and cognitive control. Biol. Psychiatry 69, e113–e125. 10.1016/j.biopsych.2011.03.028 21531388 PMC3111448

[B31] Dannemiller J. L. (1998). A competition model of exogenous orienting in 3.5-month-old infants. J. Exp. Child Psychol. 68, 169–201. 10.1006/jecp.1997.2426 9514768

[B32] Dannemiller J. L. (2004). Variations in birth weight within the normal range are related to visual orienting in infancy for boys but not for girls. Infant Behav. Dev. 27, 204–215. 10.1016/j.infbeh.2003.09.003

[B33] Daw J. Guo G. (2011). The influence of three genes on whether adolescents use contraception, USA 1994–2002. Popul. Stud. (Camb) 65, 253–271. 10.1080/00324728.2011.598942 21916669 PMC3716578

[B34] Dixon-Salazar T. J. Gleeson J. G. (2010). Genetic regulation of human brain development: lessons from Mendelian diseases. Ann. N Y Acad. Sci. 1214, 156–167. 10.1111/j.1749-6632.2010.05819.x 21062301 PMC4915827

[B35] Douet V. Chang L. Cloak C. Ernst T. (2014). Genetic influences on brain developmental trajectories on neuroimaging studies: from infancy to young adulthood. Brain Imaging Behav. 8, 234–250. 10.1007/s11682-013-9260-1 24077983 PMC3969783

[B36] Dougherty T. M. Haith M. M. (1997). Infant expectations and reaction time as predictors of childhood speed of processing and IQ. Dev. Psychol. 33, 146–155. 10.1037/0012-1649.33.1.146 9050399

[B37] Dumontheil I. Roggeman C. Ziermans T. Peyrard-Janvid M. Matsson H. Kere J.. (2011). Influence of the COMT genotype on working memory and brain activity changes during development. Biol. Psychiatry 70, 222–229. 10.1016/j.biopsych.2011.02.027 21514925

[B38] DuPaul G. J. Stoner G. D. (2014). ADHD in the Schools : Assessment and Intervention Strategies. 3rd Edn. New Yor, NY: Guilford Press.

[B39] Dye M. W. G. Bavelier D. (2010). Differential development of visual attention skills in school-age children. Vision Res. 50, 452–459. 10.1016/j.visres.2009.10.010 19836409 PMC2824025

[B40] Dye M. W. G. Hauser P. C. (2014). Sustained attention, selective attention and cognitive control in deaf and hearing children. Hear. Res. 309, 94–102. 10.1016/j.heares.2013.12.001 24355653 PMC3928356

[B41] Edwards A. C. Dodge K. A. Latendresse S. J. Lansford J. E. Bates J. E. Pettit G. S.. (2010). MAOA-uVNTR and early physical discipline interact to influence delinquent behavior. J. Child Psychol. Psychiatry 51, 679–687. 10.1111/j.1469-7610.2009.02196.x 19951362 PMC3035042

[B42] Enciso M. Sarasa J. Xanthopoulou L. Bristow S. Bowles M. Fragouli E.. (2016). Polymorphisms in the MTHFR gene influence embryo viability and the incidence of aneuploidy. Hum. Genet. 135, 555–568. 10.1007/s00439-016-1652-z 27068821

[B43] English B. A. Hahn M. K. Gizer I. R. Mazei-Robison M. Steele A. Kurnik D. M.. (2009). Choline transporter gene variation is associated with attention-deficit hyperactivity disorder. J. Neurodev. Disord. 1, 252–263. 10.1007/s11689-009-9033-8 21547719 PMC3164006

[B44] Enoch M. A. Steer C. D. Newman T. K. Gibson N. Goldman D. (2010). Early life stress, MAOA and gene-environment interactions predict behavioral disinhibition in children. Genes Brain Behav. 9, 65–74. 10.1111/j.1601-183x.2009.00535.x 19804559 PMC2824071

[B45] Eppstein M. J. Payne J. L. Goodnight C. J. (2009). Underdominance, multiscale interactions and self-organizing barriers to gene flow. J. Artif. Evol. Appl. 2009, 1–13. 10.1155/2009/725049

[B46] Erickson L. C. Thiessen E. D. Godwin K. E. Dickerson J. P. Fisher A. V. (2015). Endogenously and exogenously driven selective sustained attention: contributions to learning in kindergarten children. J. Exp. Child Psychol. 138, 126–134. 10.1016/j.jecp.2015.04.011 26044539

[B47] Ernst L. H. Lutz E. Ehlis A.-C. Fallgatter A. J. Reif A. Plichta M. M. (2013). Genetic variation in MAOA modulates prefrontal cortical regulation of approach-avoidance reactions. Neuropsychobiology 67, 168–180. 10.1159/000346582 23548774

[B48] Essex M. J. Boyce W. T. Goldstein L. H. Armstrong J. M. Kraemer H. C. Kupfer D. J.. (2002). The confluence of mental, physical, social and academic difficulties in middle childhood. II: developing the macarthur health and behavior questionnaire. J. Am. Acad. Child Adolesc. Psychiatry 41, 588–603. 10.1097/00004583-200205000-00017 12014792

[B49] Fagan J. F. III (1970). Memory in the infant. J. Exp. Child Psychol. 9, 217–226. 10.1016/0022-0965(70)90087-1 5452116

[B50] Fagan J. F. Singer L. T. (1983). Infant recognition memory as a measure of intelligence. Adv. Infancy Res. 2, 31–78.

[B51] Fantz R. (1961). The origin of human form perception. Sci. Am. 204, 66–72. 10.1038/scientificamerican0561-66 13698138

[B52] Faraone S. V. Mick E. (2010). Molecular genetics of attention deficit hyperactivity disorder. Psychiatr. Clin. North Am. 33, 159–180. 10.1016/j.psc.2009.12.004 20159345 PMC2847260

[B53] Farrington D. P. (1991). Longitudinal research strategies: advantages, problems and prospects. J. Am. Acad. Child Adolesc. Psychiatry 30, 369–374. 10.1097/00004583-199105000-00003 2055872

[B54] Feng Y. Crosbie J. Wigg K. Pathare T. Ickowicz A. Schachar R.. (2005). The SNAP25 gene as a susceptibility gene contributing to attention-deficit hyperactivity disorder. Mol. Psychiatry 10, 998–1005. 10.1038/sj.mp.4001722 16088329

[B55] Fergusson D. M. Boden J. M. Horwood L. J. Miller A. L. Kennedy M. A. (2011). MAOA, abuse exposure and antisocial behaviour: 30-year longitudinal study. Br. J. Psychiatry 198, 457–463. 10.1192/bjp.bp.110.086991 21628708 PMC3105117

[B56] Fisher S. E. Francks C. McCracken J. T. McGough J. J. Marlow A. J. MacPhie L.. (2002). A genomewide scan for loci involved in attention-deficit/ hyperactivity disorder. Am. J. Hum. Genet. 70, 1183–1196. 10.1086/340112 11923911 PMC447594

[B57] Frank Y. Pergolizzi R. G. Perilla M. J. (2004). Dopamine D4 receptor gene and attention deficit hyperactivity disorder. Pediatr. Neurol. 31, 345–348. 10.1016/j.pediatrneurol.2004.06.010 15519116

[B58] Gálvez J. M. Forero D. A. Fonseca D. J. Mateus H. E. Talero-Gutierrez C. Velez-van-Meerbeke A. (2014). Evidence of association between SNAP25 gene and attention deficit hyperactivity disorder in a Latin American sample. Atten. Defic. Hyperact. Disord. 6, 19–23. 10.1007/s12402-013-0123-9 24362847

[B59] Garrett M. C. Soares-da-Silva P. (1990). Role of type A and B monoamine oxidase on the formation of 3,4-dihydroxyphenylacetic acid (DOPAC) in tissues from the brain of the rat. Neuropharmacology 29, 875–879. 10.1016/0028-3908(90)90136-f 2123970

[B60] Genro J. P. Polanczyk G. V. Zeni C. Oliveira A. S. Roman T. Rohde L. A.. (2008). A common haplotype at the dopamine transporter gene 5′ region is associated with attention-deficit/hyperactivity disorder. Am. J. Med. Genet. B Neuropsychiatr. Genet. 147B, 1568–1575. 10.1002/ajmg.b.30863 18802919

[B61] Giedd J. N. Rapoport J. L. (2010). Structural MRI of pediatric brain development: what have we learned and where are we going? Neuron 67, 728–734. 10.1016/j.neuron.2010.08.040 20826305 PMC3285464

[B62] Gilman S. R. Chang J. Xu B. Bawa T. S. Gogos J. A. Karayiorgou M.. (2012). Diverse types of genetic variation converge on functional gene networks involved in schizophrenia. Nat. Neurosci. 15, 1723–1728. 10.1038/nn.3261 23143521 PMC3689007

[B63] Giros B. el Mestikawy S. Godinot N. Zheng K. Han H. Yang-Feng T.. (1992). Cloning, pharmacological characterization and chromosome assignment of the human dopamine transporter. Mol. Pharmacol. 42, 383–390. 10.1016/0924-977x(92)90103-f 1406597

[B64] Glück J. Indurkhya A. (2001). Assessing changes in the longitudinal salience of items within constructs. J. Adolesc. Res. 16, 169–187. 10.1177/0743558401162004

[B65] Grammer J. K. Coffman J. L. Ornstein P. A. Morrison F. J. (2013). Change over time: conducting longitudinal studies of children’s cognitive development. J. Cogn. Dev. 14, 515–528. 10.1080/15248372.2013.833925 24955035 PMC4063681

[B66] Greenwood P. M. Sunderland T. Friz J. L. Parasuraman R. (2000). Genetics and visual attention: selective deficits in healthy adult carriers of the epsilon 4 allele of the apolipoprotein E gene. Proc. Natl. Acad. Sci. U S A 97, 11661–11666. 10.1073/pnas.97.21.11661 11027364 PMC17257

[B67] Guinmarães A. P. Zeni C. Polanczyk G. Genro J. P. Roman T. Rohde L. A.. (2009). MAOA is associated with methylphenidate improvement of oppositional symptoms in boys with attention deficit hyperactivity disorder. Int. J. Neuropsychopharmacol. 12, 709–714. 10.1017/s1461145709000212 19309535

[B68] Haberstick B. C. Lessem J. M. Hewitt J. K. Smolen A. Hopfer C. J. Halpern C. T.. (2014). MAOA genotype, childhood maltreatment and their interaction in the etiology of adult antisocial behaviors. Biol. Psychiatry 75, 25–30. 10.1016/j.biopsych.2013.03.028 23726513 PMC3815496

[B69] Hardy G. H. (1908). Mendelian proportions in a mixed population. Science 28, 49–50. 10.1126/science.28.706.49 17779291

[B70] Hearst M. O. Patnode C. D. Sirard J. R. Farbakhsh K. Lytle L. A. (2012). Multilevel predictors of adolescent physical activity: a longitudinal analysis. Int. J. Behav. Nutr. Phys. Act. 9:8. 10.1186/1479-5868-9-8 22309949 PMC3305547

[B71] Henderickx D. Maetens K. Soetens E. (2010). Feature integration and spatial attention: common processes for endogenous and exogenous orienting. Psychol. Res. 74, 239–254. 10.1007/s00426-009-0251-1 19639338

[B72] Henly S. J. Wyman J. F. Gaugler J. E. (2011). Health trajectory research: a call to action for nursing science. Nurs. Res. 60, S79–S82. 10.1097/nnr.0b013e31821cc240 21543965 PMC5766261

[B73] Hikosaka O. Miyauchi S. Shimojo S. (1993). Voluntary and stimulus-induced attention detected as motion sensation. Perception 22, 517–526. 10.1068/p220517 8414878

[B74] Hill J. Breen G. Quinn J. Tibu F. Sharp H. Pickles A. (2013). Evidence for interplay between genes and maternal stress *in utero*: monoamine oxidase a polymorphism moderates effects of life events during pregnancy on infant negative emotionality at 5 weeks. Genes Brain Behav. 12, 388–396. 10.1111/gbb.12033 23480342

[B75] Holmboe K. Nemoda Z. Fearon R. M. Csibra G. Sasvari-Szekely M. Johnson M. H. (2010). Polymorphisms in dopamine system genes are associated with individual differences in attention in infancy. Dev. Psychol. 46, 404–416. 10.1037/a0018180 20210499 PMC3276838

[B76] Hotamisligil G. S. Breakefield X. O. (1991). Human monoamine oxidase a gene determines levels of enzyme activity. Am. J. Hum. Genet. 49, 383–392. 1678250 PMC1683299

[B77] Jacobs B. L. Azmitia E. C. (1992). Structure and function of the brain serotonin system. Physiol. Rev. 72, 165–229. 1731370 10.1152/physrev.1992.72.1.165

[B78] Juckel G. Schumacher C. Giegling I. Assion H.-J. Mavrogiorgou P. Pogarell O.. (2010). Serotonergic functioning as measured by the loudness dependence of auditory evoked potentials is related to a haplotype in the brain-derived neurotrophic factor (BDNF) gene. J. Psychiatr. Res. 44, 541–546. 10.1016/j.jpsychires.2009.11.006 20004415

[B79] Kaufman S. B. Reynolds M. R. Liu X. Kaufman A. S. McGrew K. S. (2012). Are cognitive “g” and academic achievement “g” one and the same “g”? An exploration on the Woodcock-Johnson and Kaufman tests. Intelligence 40, 123–138. 10.1016/j.intell.2012.01.009

[B80] Kavsek M. (2004). Predicting later IQ from infant visual habituation and dishabituation: a meta-analysis. J. Appl. Dev. Psychol. 25, 369–393. 10.1016/j.appdev.2004.04.006

[B81] Konrad K. Neufang S. Thiel C. M. Specht K. Hanisch C. Fan J.. (2005). Development of attentional networks: an fMRI study with children and adults. Neuroimage 28, 429–439. 10.1016/j.neuroimage.2005.06.065 16122945

[B82] Kopsida E. Mikaelsson M. A. Davies W. (2011). The role of imprinted genes in mediating susceptibility to neuropsychiatric disorders. Horm. Behav. 59, 375–382. 10.1016/j.yhbeh.2010.04.005 20403360

[B83] Lambe E. K. Fillman S. G. Webster M. J. Weickert C. S. (2011). Serotonin receptor expression in human prefrontal cortex: balancing excitation and inhibition across postnatal development. PLoS One 6:e22799. 10.1371/journal.pone.0022799 21829518 PMC3146513

[B84] Lavigne J. V. Gouze K. R. Bryant F. B. Hopkins J. (2014). Dimensions of oppositional defiant disorder in young children: heterotypic continuity with anxiety and depression. J. Abnorm. Child Psychol. 42, 937–951. 10.1007/s10802-014-9853-1 24497230 PMC4090253

[B86] Lawson H. A. Cady J. E. Partridge C. Wolf J. B. Semenkovich C. F. Cheverud J. M. (2011). Genetic effects at pleiotropic loci are context- dependent with consequences for the maintenance of genetic variation in populations. PLoS Genet. 7:e1002256. 10.1371/journal.pgen.1002256 21931559 PMC3169520

[B85] Lawson D. C. Turic D. Langley K. Pay H. M. Govan C. F. Norton N.. (2003). Association analysis of monoamine oxidase a and attention deficit hyperactivity disorder. Am. J. Med. Genet. B Neuropsychiatr. Genet. 116B, 84–89. 10.1002/ajmg.b.10002 12497620

[B87] Lee S. (2011). Deviant peer affiliation and antisocial behavior: interaction with Monoamine Oxidase A (MAOA) genotype. J. Abnorm. Child Psychol. 39, 321–332. 10.1007/s10802-010-9474-2 21152968 PMC3066389

[B88] Lee Y. Styne D. (2013). Influences on the onset and tempo of puberty in human beings and implications for adolescent psychological development. Horm. Behav. 64, 250–261. 10.1016/j.yhbeh.2013.03.014 23998669

[B89] Lemery-Chalfant K. Doelger L. Goldsmith H. H. (2008). Genetic relations between effortful and attentional control and symptoms of psychopathology in middle childhood. Infant Child Dev. 17, 365–385. 10.1002/icd.581 27076792 PMC4828044

[B91] Li D. Sham P. C. Owen M. J. He L. (2006). Meta-analysis shows significant association between dopamine system genes and attention deficit hyperactivity disorder (ADHD). Hum. Mol. Genet. 15, 2276–2284. 10.1093/hmg/ddl152 16774975

[B92] Lowe N. Kirley A. Mullins C. Fitzgerald M. Gill M. Hawi Z. (2004). Multiple marker analysis at the promoter region of the DRD4 gene and ADHD: evidence of linkage and association with the SNP -616. Am. J. Med. Genet. B Neuropsychiatr. Genet. 131B, 33–37. 10.1002/ajmg.b.30071 15389764

[B93] Lundwall R. A. (2013). Molecular Genetics and the Development of Reflexive Visual Attention (PhD). Houston, TX: Rice University Available online at: https://scholarship.rice.edu/handle/1911/76701

[B94] Macaluso E. Doricchi F. (2013). Attention and predictions: control of spatial attention beyond the endogenous-exogenous dichotomy. Front. Hum. Neurosci. 7:685. 10.3389/fnhum.2013.00685 24155707 PMC3800774

[B95] Manolakes G. Sheldon W. D. (1955). Relation between reading-test scores and language-factors intelligence quotients. Elem. Sch. J. 55, 346–350. 10.1086/458698

[B96] Manor I. (2002). Family-based and association studies of monoamine oxidase a and attention deficit hyperactivity disorder (ADHD): Preferential transmission of the long promoter-region repeat and its association with impaired performance on a continuous performance test (TOVA). Mol. Psychiatry 7, 626–632. 10.1038/sj.mp.4001037 12140786

[B97] Manuck S. B. Flory J. D. Ferrell R. E. Mann J. J. Muldoon M. F. (2000). A regulatory polymorphism of the monoamine oxidase-A gene may be associated with variability in aggression, impulsivity and central nervous system serotonergic responsivity. Psychiatry Res. 95, 9–23. 10.1016/s0165-1781(00)00162-1 10904119

[B98] Markant J. Cicchetti D. Hetzel S. Thomas K. M. (2014). Relating dopaminergic and cholinergic polymorphisms to spatial attention in infancy. Dev. Psychol. 50, 360–369. 10.1037/a0033172 23731290 PMC4034391

[B99] Marx I. Hübner T. Herpertz S. C. Berger C. Reuter E. Kircher T.. (2010). Cross-sectional evaluation of cognitive functioning in children, adolescents and young adults with ADHD. J. Neural Transm. (Vienna) 117, 403–419. 10.1007/s00702-009-0345-3 19953279

[B100] McCall R. B. (1994). What process mediates predictions of childhood IQ from infant habituation and recognition memory? Speculations on the roles of inhibition and rate of information processing. Intelligence 18, 107–125. 10.1016/0160-2896(94)90022-1

[B101] McKinnon L. A. Nathanson N. M. (1995). Tissue-specific regulation of muscarinic acetylcholine receptor expression during embryonic development. J. Biol. Chem. 270, 20636–20642. 10.1074/jbc.270.35.20636 7657643

[B102] Meck W. H. Benson A. M. (2002). Dissecting the brain’s internal clock: how frontal-striatal circuitry keeps time and shifts attention. Brain Cogn. 48, 195–211. 10.1006/brcg.2001.1313 11812042

[B103] Melendres M. C. Lutz J. M. Rubin E. D. Marcus C. L. (2004). Daytime sleepiness and hyperactivity in children with suspected sleep-disordered breathing. Pediatrics 114, 768–775. 10.1542/peds.2004-0730 15342852

[B104] Miller J. L. Vaillancourt T. Boyle M. H. (2009). Examining the heterotypic continuity of aggression using teacher reports: results from a national Canadian study. Soc. Dev. 18, 164–180. 10.1111/j.1467-9507.2008.00480.x

[B105] Moss H. A. Hess R. Swift C. F. (1982). Early Intervention Programs for Infants. New York, NY: Haworth Press.

[B106] Mueller S. C. Hardin M. G. Mogg K. Benson V. Bradley B. P. Reinholdt-Dunne M. L.. (2012). The influence of emotional stimuli on attention orienting and inhibitory control in pediatric anxiety. J. Child Psychol. Psychiatry 53, 856–863. 10.1111/j.1469-7610.2012.02541.x 22409260 PMC3427735

[B107] Murray K. Rieger E. Byrne D. (2013). A longitudinal investigation of the mediating role of self-esteem and body importance in the relationship between stress and body dissatisfaction in adolescent females and males. Body Image 10, 544–551. 10.1016/j.bodyim.2013.07.011 23993480

[B108] Nair V. D. Mishra R. K. (1995). Ontogenic development of dopamine D4 receptor in rat brain. Brain Res. Dev. Brain Res. 90, 180–183. 10.1016/0165-3806(96)83499-7 8719342

[B109] Nativio R. Sparago A. Ito Y. Weksberg R. Riccio A. Murrell A. (2011). Disruption of genomic neighbourhood at the imprinted IGF2–H19 locus in Beckwith-Wiedemann syndrome and Silver-Russell syndrome. Hum. Mol. Genet. 20, 1363–1374. 10.1093/hmg/ddr018 21282187 PMC3049359

[B110] Neumann S. A. Lawrence E. C. Jennings J. R. Ferrell R. E. Manuck S. B. (2005). Heart rate variability is associated with polymorphic variation in the cholin transporter gene. Psychosom. Med. 67, 168–171. 10.1097/01.psy.0000155671.90861.c2 15784779

[B111] Nicholson R. M. Leiter M. P. Laschinger H. K. S. (2014). Predicting cynicism as a function of trust and civility: a longitudinal analysis. J. Nurs. Manag. 22, 974–983. 10.1111/jonm.12073 23607579

[B112] Nobile M. Rusconi M. Bellina M. Marino C. Giorda R. Carlet O.. (2010). COMT Val158Met polymorphism and socioeconomic status interact to predict attention deficit/hyperactivity problems in children aged 10–14. Eur. Child Adolesc. Psychiatry 19, 549–557. 10.1007/s00787-009-0080-1 19946720

[B113] Normand S. Flora D. B. Toplak M. E. Tannock R. (2012). Evidence for a general ADHD factor from a longitudinal general school population study. J. Abnorm. Child Psychol. 40, 555–567. 10.1007/s10802-011-9584-5 22033884

[B114] Nymberg C. Jia T. Lubbe S. Ruggeri B. Desrivieres S. Barker G.. (2013). Neural mechanisms of attention-deficit/hyperactivity disorder symptoms are stratified by MAOA genotype. Biol. Psychiatry 74, 607–614. 10.1016/j.biopsych.2013.03.027 23746540

[B115] Oades R. D. Lasky-Su J. Christiansen H. Faraone S. V. Sonuga-Barke E. J. Banaschewski T.. (2008). The influence of serotonin- and other genes on impulsive behavioral aggression and cognitive impulsivity in children with attention-deficit/hyperactivity disorder (ADHD): findings from a family-based association test (FBAT) analysis. Behav. Brain Funct. 4:48. 10.1186/1744-9081-4-48 18937842 PMC2577091

[B116] Obias J. D. Lowe S. Holcomb G. W. (1992). Anesthetic considerations of an infant with Beckwith-Wiedemann syndrome. J. Clin. Anesth. 4, 484–486. 10.1016/0952-8180(92)90224-o 1457118

[B117] O’Fallon B. Adler F. R. (2006). Stochasticity, complex spatial structure and the feasibility of the shifting balance theory. Evolution 60, 448–459. 10.1554/05-403.1 16637490

[B118] Oriá R. B. Patrick P. D. Oriá M. O. B. Lorntz B. Thompson M. R. Azevedo O. G. R.. (2010). ApoE polymorphisms and diarrheal outcomes in Brazilian shanty town children. Braz. J. Med. Biol. Res. 43, 249–256. 10.1590/s0100-879x2010007500003 20401432 PMC3057459

[B119] Parasuraman R. Greenwood P. M. Sunderland T. (2002). The apolipoprotein E gene, attention and brain function. Neuropsychology 16, 254–274. 10.1037/0894-4105.16.2.254 11949718 PMC1350934

[B120] Parikh V. Naughton S. Yegla B. Guzman D. (2016). Impact of partial dopamine depletion on cognitive flexibility in BDNF heterozygous mice. Psychopharmacology (Berl) 233, 1361–1375. 10.1007/s00213-016-4229-6 26861892 PMC4814303

[B121] Peischl S. Bürger R. (2008). Evolution of dominance under frequency-dependent intraspecific competition. J. Theor. Biol. 251, 210–226. 10.1016/j.jtbi.2007.11.014 18177673

[B122] Petersen I. T. Bates J. E. Dodge K. A. Lansford J. E. Pettit G. S. (2015). Describing and predicting developmental profiles of externalizing problems from childhood to adulthood. Dev. Psychopathol. 27, 791–818. 10.1017/s0954579414000789 25166430 PMC4344932

[B123] Pickles A. Hill J. Breen G. Quinn J. Abbott K. Jones H.. (2013). Evidence for interplay between genes and parenting on infant temperament in the first year of life: monoamine oxidase a polymorphism moderates effects of maternal sensitivity on infant anger proneness. J. Child Psychol. Psychiatry 54, 1308–1317. 10.1111/jcpp.12081 23738520

[B124] Pierce K. Conant D. Hazin R. Stoner R. Desmond J. (2011). Preference for geometric patterns early in life as a risk factor for autism. Arch. Gen. Psychiatry 68, 101–109. 10.1001/archgenpsychiatry.2010.113 20819977 PMC4894313

[B125] Piton A. Poquet H. Redin C. Masurel A. Lauer J. Muller J.. (2014). 20 ans après: a second mutation in MAOA identified by targeted high-throughput sequencing in a family with altered behavior and cognition. Eur. J. Hum. Genet. 22, 776–783. 10.1038/ejhg.2013.243 24169519 PMC4023218

[B126] Plomin R. Emde R. N. Braungart J. M. Campos J. Corley R. Fulker D. W.. (1993). Genetic change and continuity from fourteen to twenty months: the MacArthur Longitudinal Twin study. Child Dev. 64, 1354–1376. 10.2307/1131539 8222877

[B127] Poirier J. (1996). Apolipoprotein E in the brain and its role in Alzheimer’s disease. J. Psychiatry Neurosci. 21, 128–134. 8820179 PMC1188752

[B128] Priess-Groben H. A. Hyde J. S. (2013). 5-HTTLPR X stress in adolescent depression: moderation by MAOA and gender. J. Abnorm. Child Psychol. 41, 281–294. 10.1007/s10802-012-9672-1 22836288

[B129] Putnam S. P. Rothbart M. K. Gartstein M. A. (2008). Homotypic and heterotypic continuity of fine-grained temperament during infancy, toddlerhood and early childhood. Infant Child Dev. 17, 387–405. 10.1002/icd.582

[B130] Razgado-Hernandez L. F. Espadas-Alvarez A. J. Reyna-Velazquez P. Sierra-Sanchez A. Anaya-Martinez V. Jimenez-Estrada I.. (2015). The transfection of BDNF to dopamine neurons potentiates the effect of dopamine D3 receptor agonist recovering the striatal innervation, dendritic spines and motor behavior in an aged rat model of Parkinson’s disease. PLoS One 10:e0117391. 10.1371/journal.pone.0117391 25693197 PMC4332861

[B131] Ribasés M. Hervás A. Ramos-Quiroga J. A. Bosch R. Bielsa A. Gastaminza X.. (2008). Association study of 10 genes encoding neurotrophic factors and their receptors in adult and child attention-deficit/hyperactivity disorder. Biol. Psychiatry 63, 935–945. 10.1016/j.biopsych.2007.11.004 18179783

[B132] Riikonen R. S. Jääskeläinen J. Turpeinen U. (2010). Insulin-like growth factor-1 is associated with cognitive outcome in infantile spasms. Epilepsia 51, 1283–1289. 10.1111/j.1528-1167.2009.02499.x 20163445

[B133] Rommelse N. N. Altink M. E. Arias-Vásquez A. Buschgens C. J. Fliers E. Faraone S. V.. (2008). A review and analysis of the relationship between neuropsychological measures and DAT1 in ADHD. Am. J. Med. Genet. B Neuropsychiatr. Genet. 147B, 1536–1546. 10.1002/ajmg.b.30848 18729135

[B134] Rose S. A. Feldman J. F. (1997). Memory and speed: their role in the relation of infant information processing to later IQ. Child Dev. 68, 630–641. 10.2307/1132115 9306643

[B136] Rose S. A. Feldman J. F. Jankowski J. J. (2003). Infant visual recognition memory: independent contributions of speed and attention. Dev. Psychol. 39, 563–571. 10.1037/0012-1649.39.3.563 12760523

[B135] Rose S. A. Wallace I. F. (1985). Visual recognition memory: a predictor of later cognitive functioning in preterms. Child Dev. 56, 843–852. 10.2307/1130096 4042748

[B137] Saarenpää-Heikkilä O. Laippala P. Koivikko M. (2000). Subjective daytime sleepiness in schoolchildren. Fam. Pract. 17, 129–133. 10.1093/fampra/17.2.129 10758074

[B138] Scherbaum C. A. Ferreter J. M. (2009). Estimating statistical power and required sample sizes for organizational research using multilevel modeling. Organ. Res. Methods 12, 347–367. 10.1177/1094428107308906

[B139] Seif I. De Maeyer E. (1999). Knockout corner: knockout mice for monoamine oxidase A. Int. J. Neuropsychopharmacol. 2, 241–243. 10.1017/s1461145799001534 11281992

[B140] Selig J. P. Little T. D. (2012). “Autoregressive and cross-lagged panel analysis for longitudinal data,” in Handbook of Developmental Research Methods, eds Laursen B. Little T. D. Card N. A. (New York, NY: Guilford Press), 265–278.

[B141] Shackleton T. M. Skottun B. C. Arnott R. H. Palmer A. R. (2003). Interaural time difference discrimination thresholds for single neurons in the inferior colliculus of Guinea pigs. J. Neurosci. 23, 716–724. 12533632 10.1523/JNEUROSCI.23-02-00716.2003 PMC6741888

[B142] Shaw P. Gornick M. Lerch J. Addington A. Seal J. Greenstein D.. (2007). Polymorphisms of the dopamine D4 receptor, clinical outcome and cortical structure in attention-deficit/hyperactivity disorder. Arch. Gen. Psychiatry 64, 921–931. 10.1001/archpsyc.64.8.921 17679637

[B143] Shim S. H. Hwangbo Y. Kwon Y. J. Jeong H. Y. Lee B. H. Lee H. J.. (2008). Increased levels of plasma brain-derived neurotrophic factor (BDNF) in children with attention deficit-hyperactivity disorder (ADHD). Prog. Neuropsychopharmacol. Biol. Psychiatry 32, 1824–1828. 10.1016/j.pnpbp.2008.08.005 18760321

[B144] Sigman M. Cohen S. E. Beckwith L. (1997). Why does infant attention predict adolescent intelligence? Infant Behav. Dev. 20, 133–140. 10.1016/s0163-6383(97)90016-3

[B145] Smith K. M. Daly M. Fischer M. Yiannoutsos C. T. Bauer L. Barkley R.. (2003). Association of the dopamine beta hydroxylase gene with attention deficit hyperactivity disorder: genetic analysis of the Milwaukee longitudinal study. Am. J. Med. Genet. B Neuropsychiatr. Genet. 119B, 77–85. 10.1002/ajmg.b.20005 12707943

[B146] Störmer V. S. Passow S. Biesenack J. Li S. C. (2012). Dopaminergic and cholinergic modulations of visual-spatial attention and working memory: insights from molecular genetic research and implications for adult cognitive development. Dev. Psychol. 48, 875–889. 10.1037/a0026198 22103306

[B147] Suzuki S. Cavanagh P. (1997). Focused attention distorts visual space: an attentional repulsion effect. J. Exp. Psychol. Hum. Percept. Perform. 23, 443–463. 10.1037/0096-1523.23.2.443 9104004

[B148] Teller D. Y. (1997). First glances: the vision of infants. Invest. Ophthalmol. Vis. Sci. 38, 2183–2203. 9344342

[B149] Torgersen A. M. (1981). Genetic factors in temperamental individuality: a longitudinal study of same-sexed twins from two months to six years of age. J. Am. Acad. Child Psychiatry 20, 702–711. 10.1097/00004583-198102000-00003 7199060

[B150] Tucker-Drob E. M. Briley D. A. Harden K. P. (2013). Genetic and environmental influences on cognition across development and context. Curr. Dir. Psychol. Sci. 22, 349–355. 10.1177/0963721413485087 24799770 PMC4006996

[B151] Underbjerg M. George M. S. Thorsen P. Kesmodel U. S. Mortensen E. L. Manly T. (2013). Separable sustained and selective attention factors are apparent in 5-year-old children. PLoS One 8:e82843. 10.1371/journal.pone.0082843 24376591 PMC3869710

[B152] Voigt G. Montag C. Markett S. Reuter M. (2015). On the genetics of loss aversion: an interaction effect of BDNF Val66Met and DRD2/ANKK1 Taq1a. Behav. Neurosci. 129, 801–811. 10.1037/bne0000102 26501178

[B153] Walitza S. Renner T. J. Dempfle A. Konrad K. Wewetzer C. Halbach A.. (2005). Transmission disequilibrium of polymorphic variants in the tryptophan hydroxylase-2 gene in attention-deficit/hyperactivity disorder. Mol. Psychiatry 10, 1126–1132. 10.1038/sj.mp.4001734 16116490

[B154] Wargelius H.-L. Malmberg K. Larsson J.-O. Oreland L. (2012). Associations of MAOA-VNTR or 5HTT-LPR alleles with attention-deficit hyperactivity disorder symptoms are moderated by platelet monoamine oxidase B activity. Psychiatr. Genet. 22, 42–45. 10.1097/ypg.0b013e328347c1ab 21610556

[B155] Webb S. J. Jones E. J. H. (2009). Early identification of autism: early characteristics, onset of symptoms and diagnostic stability. Infants Young Child. 22, 100–118. 10.1097/iyc.0b013e3181a02f7f PMC5232420 28090148

[B156] Whelan Y. M. Kretschmet T. Barker E. D. (2014). MAOA, early experiences of harsh parenting, irritable opposition and bullying-victimization: a moderated indirect-effects analysis. Merrill Palmer Q. 60, 217–237. 10.13110/merrpalmquar1982.60.2.0217

[B157] Wilson D. S. Turelli M. (1986). Stable underdominance and the evolutionary invasion of empty niches. Am. Nat. 127, 835–850. 10.1086/284528

[B158] Winterer G. Musso F. Konrad A. Vucurevic G. Stoeter P. Sander T.. (2007). Association of attentional network function with exon 5 variations of the CHRNA4 gene. Hum. Mol. Genet. 16, 2165–2174. 10.1093/hmg/ddm168 17613539

[B159] Xu X. Brookes K. Chen C. K. Huang Y. S. Wu Y. Y. Asherson P. (2007). Association study between the monoamine oxidase A gene and attention deficit hyperactivity disorder in Taiwanese samples. BMC Psychiatry 7:10. 10.1186/1471-244x-7-10 17328795 PMC1810533

[B160] Yu Y. W.-Y. Tsai S.-J. Hong C.-J. Chen M.-C. Yang C.-W. Chen T.-J. (2005). Association study of a functional MAOA-uVNTR gene polymorphism and cognitive function in healthy females. Neuropsychobiology 52, 77–82. 10.1159/000086609 15990460

[B161] Zhang J. Chen Y. Zhang K. Yang H. Sun Y. Fang Y.. (2010). A cis-phase interaction study of genetic variants within the MAOA gene in major depressive disorder. Biol. Psychiatry 68, 795–800. 10.1016/j.biopsych.2010.06.004 20691428

[B162] Ziats M. N. Rennert O. M. (2011). Expression profiling of autism candidate genes during human brain development implicates central immune signaling pathways. PLoS One 6:e24691. 10.1371/journal.pone.0024691 21935439 PMC3174192

[B163] Zohsel K. Bianchi V. Mascheretti S. Hohm E. Schmidt M. H. Esser G.. (2015). Monoamine oxidase a polymorphism moderates stability of attention problems and susceptibility to life stress during adolescence. Genes Brain Behav. 14, 565–572. 10.1111/gbb.12258 26449393

